# Supplementary material for: Operative and Technical Modifications to the Coriolis® µ Air Sampler That Improve Sample Recovery and Biosafety During Microbiological Air Sampling
Source: Ann Work Expo Health. 2020 May 29;64(8):852–65. doi: 10.1093/annweh/wxaa053 (PMC7544001; doi:10.1093/annweh/wxaa053)
Supplement: wxaa053_suppl_Supplementary_Material [file wxaa053_suppl_supplementary_material.pdf]

SUPPLEMENTARY MATERIAL

Operative and technical modifications to the Coriolis® µ air sampler that improve sample recovery and biosafety during microbiological air sampling

Nuno Rufino de Sousa, Lei Shen, David Silcott, Charles J. Call,
Antonio Gigliotti Rothfuchs

Inventory of Supplementary material

Supplementary Figure 1, related to Figure 1. Parts description for Coriolis HEPA adaptation.

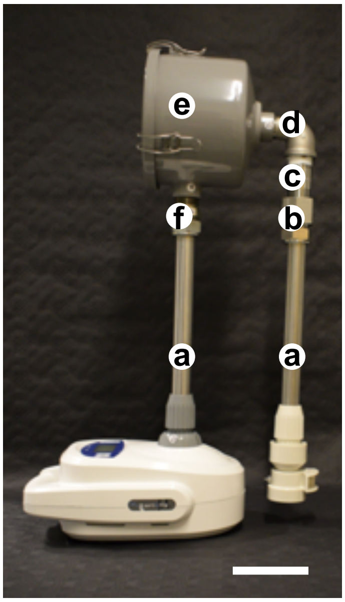

Position	Part	Part nr
a	stainless steel tubing	8989K848
b	yor-lok fitting	5182K832
	yor-lok sleeve	5182K511
c	pipe nipple	4830K225
d	threaded pipe fitting	4464K41
e	HEPA-filter casing	51685K84
	HEPA filter	9179K16
f	yor-lok fitting	5929K38
	yor-lok sleeve	5182K511

Supplementary Figure 1. Parts description for Coriolis HEPA adaptation. Photograph and parts description including catalog number (all MacMaster-Carr, USA) for assembly of HEPA adaptation, amounting in all to about 745 USD (2020). Scale bar depicting 10 cm.
